# Development and validation of a novel corpus of 269 verb pictures in Kannada based on an argument structure taxonomy

**DOI:** 10.3758/s13428-021-01662-x

**Published:** 2021-12-15

**Authors:** Wasim Ahmed, P. P. Giridhar, Gopee Krishnan

**Affiliations:** 1 Department of Speech and Hearing, Manipal College of Health Professions, Manipal Academy of Higher Education (MAHE), Manipal, India; 2 Central Institute of Indian Languages (CIIL), Mysore, India

**Keywords:** Verbs, Action pictures, Psychometric, Normative, Norms, Name agreement, Image agreement, Familiarity, Visual complexity

## Abstract

Pictorial stimuli are crucial in psycholinguistic research and clinical practice. The development of culturally and linguistically appropriate, standardized picture corpora is a tedious and meticulous process. Yet, such readily accessible picture sets are useful for researchers and clinicians alike. The current study introduces a novel set of 269 verb pictures for an Indian language – Kannada. The included verbs were selected from a published database of 100,000 words along with their frequency scores in this language, and were subsequently categorized based on an argument structure taxonomy. Each picture is developed based on an exemplar sentence that depicts a scenario rather than a mere action. Norms are provided for verb name and argument agreement, image agreement, concept familiarity, and visual complexity, along with the orthographic frequency. Correlations between these measures are also described. The complete set of pictures are freely downloadable from https://osf.io/uk2af/?view_only=ecffbd92f48546a484c869b3f0b8ec94 for academic, research, and clinical usage in the future.

## Introduction

Any standardized picture corpus is an essential tool for experimental linguistic research and clinical practice (Szekely et al., 2004). The availability of a cross-culturally and cross-linguistically analogous stimulus sets is beneficial while assessing language comprehension and production (Khwaileh et al., 2018). Further, such standardized sets of stimuli can foster the designing of comprehensive assessment and therapeutic protocols. For decades, pictures have been utilized as fundamental stimuli in psycholinguistic and neurolinguistic research. In any language, the unavailability of a universally applicable picture corpus is a serious disadvantage to experimenters who intend to conduct studies comparable with those in other languages. Consequently, experimenters and clinicians are forced to develop their own set of stimuli considering the linguistic and cultural differences, thus rendering the results of such studies incomparable (Ahmed, Lakshmanan, & Priyanka, 2013a; Ahmed, Murthy, et al., 2013b; Bonin et al., 2003; Khwaileh et al., 2018; Kremin et al., 2003; Sanfeliu & Fernandez, 1996; Tsaparina et al., 2011). These sets are seldom used beyond the realm of the experiment, test, or therapeutic program for which they are created. Thus, the demand for linguistically and culturally exclusive normative databases has increased (Khwaileh et al., 2018). Since the publication of the renowned Snodgrass and Vanderwart (1980) noun picture corpus, numerous adaptations of the same have been made into various languages (e.g., Spanish (Sanfeliu & Fernandez, 1996), British English (Barry et al., 1997), French (Alario & Ferrand, 1999), Icelandic (Pind et al., 2000), Italian (Nisi & Longoni, 2000), Portuguese (Pompéia et al., 2001), Japanese (Nishimoto et al., 2005), Chinese (Weekes et al., 2007), Modern Greek (Dimitropoulou et al., 2009), Russian (Tsaparina et al., 2011), and Tamil (Ahmed, Lakshmanan, & Priyanka, 2013a)). Further, the norms for the same corpus in children (e.g., American English (Berman et al., 1989; Cycowicz et al., 1997), French (Cannard et al., 2005), Kannada (Ahmed, Murthy, et al., 2013b), Chinese (Wang et al., 2014), and Thai (Clarke & Ludington, 2018)), and extended versions of this database are published in various languages (e.g., English (Cycowicz et al., 1997), Italian (Barca et al., 2002), French (Bonin et al., 2003), Portuguese (Cameirao & Vicente, 2010), and Turkish (Raman et al., 2014)).

Normative databases are more commonly available for nouns in comparison to verbs. These sets are frequently used in picture-naming studies. On the contrary, verb-based picture sets are predominantly developed to assess action-naming, and are sparsely available to researchers and clinicians. Among the previously developed databases, Fiez and Tranel (1997) provided norms for 280 English verbs depicted in colored photographs. Masterson and Druks (1998) offered normative data based on multiple parameters for 102 action pictures in English. This set was later normed in Spanish by Cuetos and Alija (2003). Imageability rating norms for 427 English verbs were provided by Chiarello, Shears and Lund (1999) as a part of their study to examine verb–noun dissociation. Bird, Franklin and Howard (2001) documented age of acquisition and imageability norms for an enormous picture corpus of 2645 words, which constituted 892 English verbs. An extensive multilingual normative database was provided for a corpus that contained 275 action pictures (Szekely et al., 2004). Norms are provided in seven languages, i.e., American English, Bulgarian, Italian, German, Hungarian, Mexican and Mandarin Chinese (Taiwan version). Bonin et al. (2004) provided French norms for 142 colored photographs initially developed by Fiez and Tranel (1997). Schwitter et al. (2004) offered French normative data for black-and-white line drawings, out of which 71 images belonged to the naming battery developed by Masterson and Druks (1998), and 41 drawings were additionally developed by the authors. Cameirao and Vicente (2010) offered norms for age of acquisition, frequency, and word length along with multiple other psycholinguistic parameters for a Portuguese word corpus that contained 373 verbs. Dutch normative data for most of the psychometric parameters are available for 124 action pictures (Shao et al., 2014), out of which 100 images belonged to the Druks and Masterson (2000) set and the remaining 24 belonged to Konopka and Meyer (2012). More recently, normative for 375 action pictures in Russian (Akinina et al., 2015) and 141 action pictures in Gulf Arabic (Khwaileh et al., 2018) have been made available.

Verbs describe an event or state. A lexical property that characterizes the number and types of obligatory and optional participant roles is known as argument structure (Akinina et al., 2015; Thompson & Shapiro, 2005). The argument structure interacts with the syntax and plays a vital role in the proper formation of sentences. Different kinds of verbs are determined by the number of arguments or the number of participants related to the action described by them (Masterson, Druks & Gallienne, 2008).

Verbs can be grouped based on the differences in the arrangement of their argument structure and the thematic roles (Fiez & Tranel, 1997; Thompson & Shapiro, 2005). Based on the argument structure, verbs in English could be grouped into: one-, two-, and three-argument verbs (Thompson & Shapiro, 2005). One-argument verbs (e.g., bark, laugh) are also known as intransitive verbs. They have only one external argument, which may be an agent or object or experiencer. Two-argument verbs (e.g., cut, pour) assign the agent role to an external argument and the theme role to the internal argument. Finally, the three-argument verbs may carry an agent, a theme, and a goal (e.g., give, put). These features also must be present in grammatical sentences (Fiez & Tranel, 1997). For example, consider the sentences “*the plumber repairs”* and “*The boys put Max”* are both grammatically incomplete because of poor representation of the verb arguments (Thompson, 2012, p. 737). Every verb in the lexicon is encoded along with its argument structure (Thompson & Shapiro, 2007). Processing verbs can be more taxing than nouns (Akinina et al., 2015; Damasio & Tranel, 1993). Compared to nouns, verbs demand a complex grammatical encoding, as they tend to activate more extensive lemma information, thus making them difficult to retrieve (Khwaileh et al., 2018). Verbs seem less imageable and have composite exemplification compared to nouns (Mätzig et al., 2009).

Unlike nouns, verbs may not have an associated reference. Verbs have empirical embodiment only through nouns. That is, one cannot point to any *verb* in the empirical world except through nouns that embody the actions/states that verbs denote. Nouns, however, have prior empirical embodiment. The verbs foster the embodiment of nouns to the communicative world through the argument structures (Masterson, Druks & Gallienne, 2008). The variable argument structure restricts the generalization of one verb to another (Mätzig et al., 2009). Despite these salient features, the proportion of studies on properties of verbs, such as determining their argument structure, is rather meager compared to that of nouns. Most such efforts are largely limited to a few languages like English.

## The Kannada language

Kannada is the native language of the southwestern state of Karnataka, in India. It has four major dialects. It belongs to the southern branch of the Dravidian family of languages with a history dating back to c. 450 CE. (Steever, 2003, p. 129; Karnataka Government, 2020). More than 60 million native speakers, commonly known as ‘Kannadigas’, exist globally, constituting about 5% of the Indian population (Brown & Ogilvie, 2010; Government of India, 2020). Kannadigas form a significant part of the Indian diaspora, which is the largest globally (Singh, 2020).

In Kannada, verbs are qualitatively classified into three classes: ‘Agent verbs,’ ‘Experiencer verbs,’ and ‘Object verbs’ (Giridhar, 1992, p. 160). These verb types can be further classified based on the argument structure they carry as one-, two-, or three-argument verbs, with the verb class taking its name from the case role that is subjectivized either as a nominative or dative (Giridhar, 1992, p. 160). “Kannada is a left-branching, head-final language and the canonical word order is subject-object-verb(SOV) with the main verbs preceding their auxiliaries, genitives preceding their nouns, and complements preceding matrix clauses (Steever, 2003, p. 146). Its “morphology is agglutinative and primarily suffixal” (Steever, 2003, p. 131). Considering these characteristics of Kannada, a mere adaptation or translation of verb-based pictorial stimuli from a foreign language would seem inappropriate. For instance, verbs categorized under a specific valency category in one language might not necessarily hold good in another, although it could only be parametric variation within a universal frame. What are one-place verbs in English may be two-place in Kannada. To give but one example, “ ” /Kalisu/, meaning ‘teach’ in Kannada, is a two-place (or even three-place verb because the goal seems obligatory) while it is one-place in English. Thus, the sentence, “I taught for a while” is acceptable in English, while the Kannada version

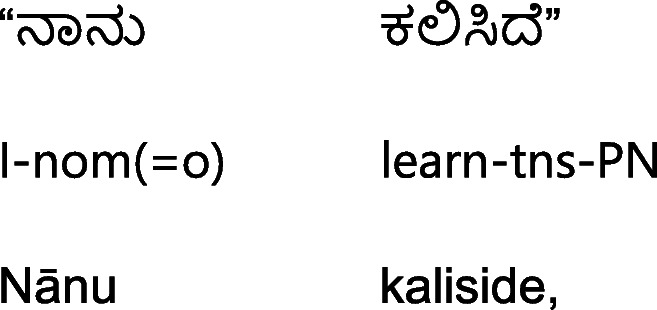



meaning “I taught” without mentioning what it is that I taught can be improper unless it is deleted because of linguistic context, which means it must have found a mention somewhere in the discourse. This is not the case in English.

Further, what are predicate adjectives in English could be a full verb in Kannada. For example, in the sentence “He is angry,” the word angry is actually a predicate adjective in English. On the contrary, in the Kannada version

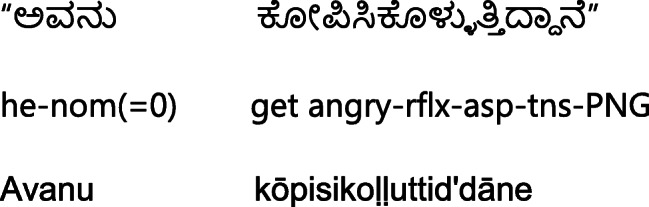



the word ‘kōpisikoḷḷuttid'dāne’ is a full verb. Thus, the development and validation of relevant stimulus set for the Kannadiga population become necessary. A standardized picture corpus needs to be linguistically and culturally apposite to enhance its validity and rigor (Khwaileh et al., 2018), especially in a multilingual country like India, where the languages are radically different from foreign languages in terms of the sentence structure. In Kannada, the availability of a normative verb dataset based on the psychometric properties is sparse and remains as a much-needed resource. Despite an overwhelming necessity and demand for such a corpus, only a handful of studies have attempted to provide such data in this language (Ahmed et al., 2013a). Most of these studies offer normative data only for nouns, but not for verbs. Hence, in this study, we aimed to develop and validate a comprehensive verb picture corpus with multiple utilities for the adult Kannadiga population. With this dataset, we intend to provide normative data based on the verb argument structure rather than mere action naming, so that a variety of thematic roles are depicted (Fiez & Tranel, 1997). We hope that the availability of such an extensive verb picture corpus would serve as a valuable asset to researchers and clinicians alike as well as would serve as a stimulus for such efforts in other languages.

## Materials and methods

This study obtained approvals from the Institutional Research Committee, Manipal College of Health Professions (MCHP) and the Kasturba Medical College and Kasturba Hospital Institutional Ethics Committee, Manipal Academy of Higher Education (MAHE), Manipal, India (IEC Registration No: 508/2018).

### Selection and classification of the picturable verbs

Two speech-language pathologists and a linguist (all Kannadigas) compiled 1104 verbs and their frequency scores from a documented corpus of 100,000 words (Ranganatha, 1982). Later, the selected verbs were classified based on their argument structure, i.e., the participant roles needed (along with a verb) to form grammatically unambiguous sentences. The authors independently assigned the argument structure to each verb, categorized them, and later compiled into a final list with consensus. For the rest of the picture developmental process, we selected a total of 300 verbs that we felt were imageable. An exemplar sentence was generated for each verb. In every sentence, the target verb and the corresponding argument structure were grammatically apposite. The exemplars represented common, natural, and unambiguous scenarios. All the exemplar sentences were reviewed and approved by a linguist, a Kannada professor, and a speech-language pathologist for cultural and linguistic appropriateness. It was ensured that the exemplar sentences did not skew towards any particular dialect of Kannada.

### Development and refinement of verb-based pictures

An artist drew images based on the exemplar sentences. The pictures were simple black outline drawings upon a white background. For each verb, the artist developed at least three prototypes. Another painting artist, a linguist, and a speech-language pathologist, voted for the best illustrations (among the prototypes) chosen to the final list. Any picture that seemed ambiguous and culturally inappropriate was drawn again and reviewed. To prevent verb name disagreement and ambiguity, pointers were used in some of the pictures to draw participants’ attention to the subject (Akinina et al., 2015; Fiez & Tranel, 1997). Subsequently, a digital artist converted each picture into a digital-colored clipart image of 1200X1200 pixels dimension. Following necessary corrections and revisions by a painting artist, a linguist, and a speech-language pathologist, a total of 269 pictures (one-argument verbs = 85; two-argument verbs = 132; three-argument verbs = 52) were included to the final list.

### Psychometric rating and validation

The newly developed verb picture corpus was unique and it categorizes verbs based on their argument structure, and provides contextually depicted pictorial representation, rather than merely naming an action. The corpus was validated using four psychometric tasks: verb name and argument agreement, image agreement, concept familiarity, and visual complexity. The procedure used for these tasks was similar to the renowned study by Snodgrass and Vanderwart (1980).

### Participants

We recruited 120 healthy adults for this study. All participants were native Kannada speakers. The participants were snowball sampled to represent four major dialects of Kannada. For each psychometric task, 30 participants were enrolled, who were further divided into three groups of ten each (with five females), across the age ranges of 18 to 40 years, 41 to 60 years, and 61 to 80 years (see Table 1). Based on the self-report, none of them had any history/complaint of cognitive, hearing, or neurological deficits. All had a minimum of 7th-grade education, and normal or corrected-to-normal vision.

**Table 1:** Mean age of participants across each psychometric task

Psychometric task	Mean age ± SD (in years)		
	18–40 years	41–60 years	61–80 years
Verb name and argument agreement	26.4 ± 5.8	49.1 ± 6.6	71.5 ± 6.3
Concept familiarity	28.7 ± 8.2	50.8 ± 5.4	70.0 ± 7.1
Visual complexity	28.8 ± 8.1	50.9 ± 5.8	69.9 ± 6.1
Image agreement	27.4 ± 7.0	51.3 ± 6.6	71.1 ± 6.0

## Procedure

All psychometric tasks were conducted via online mode during the COVID-19 pandemic lockdown phase. Each participant was requested over the telephone to participate, and on acceptance, an informed consent was obtained via e-mail. The participants were given an appointment to log in through the Google Meet platform using either a laptop or a desktop computer for a one-on-one online session. As a general procedure, each picture was displayed on the shared screen for 5 s using a Microsoft PowerPoint presentation, with a gap of 3 s between the consecutive slides. To prevent role reversal between agent and patient, pointers were used to prime the agent in some of the pictures (Akinina et al., 2015; Fiez & Tranel, 1997). Instructions for each task were displayed on the screen and orally read out to each participant. The experimenter addressed participant’s queries before commencing the task, and explained the importance of this normative study. Participants received a response sheet beforehand. They were requested to fill in their responses in the prescribed format and e-mail the response sheet to the first author. For the 71 participants (predominantly geriatric) who could not comprehend the technicalities, an option was given to speak out their responses aloud, as the experimenter noted them down. To become accustomed to the task demands, the participants received a minimum of five practice trials before each task. A 5-min break was provided halfway through the slides for all participants. The rating was completed for each participant with three sessions of 90–120 min duration, spanning across 3 days (for one-, two-, and three-argument verb sets, respectively) within a week’s time.

### Task 1 - Verb name and argument agreement

In this task, the participants (*N* = 30) were asked to observe each picture and report what was happening in it. Specific instructions were provided for the one-, two-, and three-argument verbs to encourage the participants to identify all the corresponding arguments portrayed along with the verb. Thus, in a picture with one-argument verb, the instruction was to report “Who is Doing What?”. Similarly, for the two-argument verbs, they were instructed to report “Who is Doing What to Whom/Where.” For the three-argument verbs, they had to report “Who is Doing What to Whom and Where/How.” Explicit instructions were given to avoid any single-word responses for the picture, and to encourage them to respond in a multi-word sentence form.

For example:

One-argument verb:

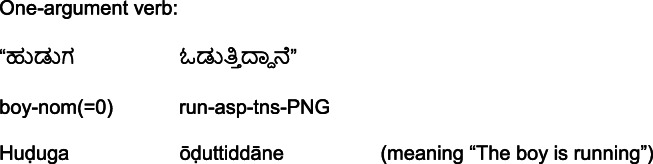



Two-argument verb:

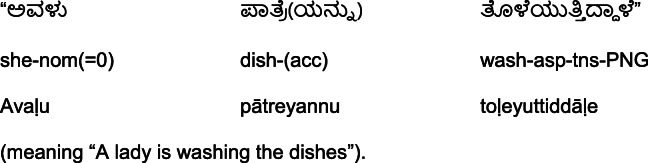



Three-argument verb:

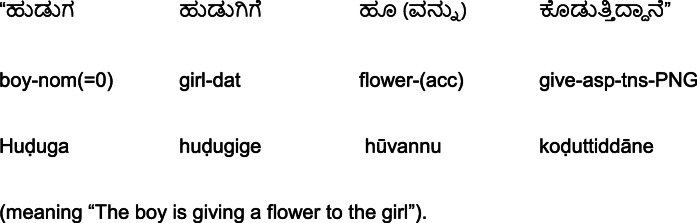



Following Snodgrass and Vanderwart (1980), any failures to identify the action(s) depicted in the pictures were also noted down. The participants had to mark ‘✘’ if they could not comprehend what was displayed in the image. If participants felt that they knew what was happening in the picture but could not recall and say/write the actual words, they had to mark ‘✓’. Those participants who chose to provide oral responses were instructed to mention either of the two directly. The responses were analyzed to identify each picture's dominant sentence, i.e., the most commonly used verb and the corresponding argument structure reported for the target image. The dominant responses were used for subsequent tasks as well as for the statistical analyses.

### Task 2 – Concept familiarity

Participants (*N* = 30) of this task were asked to judge how common each action depicted in the picture was to them in their daily lives. Participants had to judge based on how frequently they performed the action themselves or had witnessed someone else performing it. Familiarity was defined as “the degree to which an individual would think about or came in contact with the concept” (Snodgrass & Vanderwart, 1980). The experimenter read out the dominant sentence and subsequently projected the target image. A three-point rating scale (1 = ’Unfamiliar,’ 2 = ’Familiar,’ 3 = ’Very Familiar’) was also projected beside each target image to facilitate the participants' rating of the pictures. If the participants could not identify the depicted action, they had to report it to the experimenter, or mark it with ‘✘.’

### Task 3 – Visual complexity

In this psychometric task, the participants (*N* = 30) had to grade the pictures based on the extent of details or intricacy utilized to depict the verbs. A three-point rating scale (1 = ’Do Not Agree’, 2 = ’Agree’, 3 = ’Strongly Agree’) was provided to rate whether they agree that the details used in each picture were sufficient to portray it, and to identify the target verbs unambiguously as orally defined by the experimenter.

### Task 4 – Image agreement

In this task, the participants (*N* = 30) were instructed to rate the level of similitude between the displayed picture and a mental image that they had formed after listening to the target sentence uttered by the experimenter. A blank white screen was presented while the experimenter read out each sentence. In the following ~5-s blank duration, the participants formed a mental image and subsequently matched it with the then displayed target picture. A three-point rating scale (1 = ’Not Matching’, 2 = ’Moderately Matching’, 3 = ’Highly Matching’) was provided. If the participants could not generate a mental image, they had to either write ‘ ’ (‘*illa*’ meaning ‘No’ in Kannada) in the response sheets or verbally confirm it before the target picture was displayed.

### Statistical measures

For the verb name and argument agreement data, participants’ responses were grouped as either dominant or alternative names. Both percentage scores and the *H* information statistic (see below) were computed for each picture to analyze the verb name agreement scores. The *H* information statistic captures more information than the percentage scores (Akinina et al., 2015; Fiez & Tranel, 1997; Snodgrass & Vanderwart, 1980). When two pictures receive an equal percentage score, the picture that obtains lesser alternative names (*H* closer to 0.00) would have a higher name agreement (Pompéia et al., 2001; Snodgrass & Vanderwart, 1980).

*H* is defined as:

$$ \boldsymbol{H}=\sum \limits_{\boldsymbol{i}=\mathbf{1}}^{\boldsymbol{k}}{\mathbf{p}}_{\mathbf{i}}{\mathbf{\log}}_{\mathbf{2}}\left(\mathbf{1}/\mathbf{pi}\right) $$

where, ‘k’ represents the number of diverse action names received by each picture, and ‘p_i_’ denotes the proportion of the participants who provided each action name. Naming failures were included only during percentage computation. For all the four psychometric ratings, descriptive statistics along with Spearman rho correlation were computed. All statistical analyses were carried out with SPSS (version 16.0) program for Windows.

## Results and discussion

The data from one-, two-, and three-argument verbs are presented separately for easier cross-referencing. The dominant response for each picture (with transliteration, translation, IPA and Kannada transcript) along with the percentage, *H* statistic mean and standard deviation scores are provided in the appendix which can be accessed through the link: https://osf.io/uk2af/?view_only=ecffbd92f48546a484c869b3f0b8ec94

## One-argument verbs

### Name agreement

Out of the total 85 pictures developed in this subset (Table 2), 51 obtained a perfect name agreement (Pct = 100%, *H* = 0). Ten pictures received a good name agreement (*H* ≤ 0.5, Pct ≥ 85%), 22 obtained the name agreement (*H*) between 0.5 and 1.5 (Pct ≥ 60%), and the remaining two pictures’ name agreement scores were greater than 1.5 (Pct ≤ 50%). Overall, the *H* statistic, being positively skewed (0.96) with a low mean (M = 0.35; SD = 0.47) in comparison to the negatively skewed percentage scores (– 1.47) with a higher mean (M = 89.34; SD = 15.92), signifies that a majority of the pictures within this set showed a high name agreement. This is supported by the *H* statistic of 74 pictures that were lesser than the 75th percentile. However, percentage scores of 20 pictures were less than the 25th percentile.

**Table 2:** Descriptive statistics: one-argument verbs

		Name agreement		Word frequency	Concept familiarity	Visual complexity	Image agreement
		Percentage	*H* statistic				
**Mean**		89.34	0.35	5.00	2.92	2.84	2.78
**Median**		100.00	0.00	1.00	2.93	2.93	2.86
**Standard Deviation**		15.92	0.47	11.17	0.10	0.23	0.24
**Minimum**		26.66	0.00	1.00	2.46	1.80	1.93
**Maximum**		100.00	1.56	95.00	3.00	3.00	3.00
**Range**		73.33	1.56	94.00	0.53	1.20	1.06
**Skewness**		– 1.47	0.96	6.51	– 2.14	– 2.37	– 1.70
**Kurtosis**		1.82	– 0.52	50.14	5.45	6.56	2.62
**Percentile**	**25**	80.00	0.0	1.00	2.86	2.80	2.73
	**75**	100.00	0.83	5.00	3.00	3.00	2.93
**Interquartile Range**		20.00	0.83	4.00	0.13	0.20	0.20

### Familiarity and visual complexity

The participants seemed to be familiar with a majority of the concepts depicted in this set as the data was leptokurtic (5.45), and negatively skewed (– 2.14) with a high mean score (M = 2.92; SD = 0.10). Only 12 pictures obtained lesser than the 25th percentile score. Similarly, the data from visual complexity rating showed a higher mean (M = 2.83; SD = 0.23), with a leptokurtic (6.56) and negatively skewed (– 2.37) distribution suggesting that the details used to depict the concepts were adequate. However, 19 pictures received marginally lesser score than the 25th percentile.

### Image agreement

Data from this task indicated that most of the pictures from this set elicited a consistent mental image across the participants. The platykurtic (2.62), negatively skewed (– 1.70) distribution with a higher mean (M = 2.78; SD = 0.24) showed that the majority of the pictures are suitably developed to portray the target verbs. Most of the pictures were rated to match well with their respective mental images as pictures received ratings within the interquartile range (median = 2.86; IQR = 2.73– 2.93). Yet, 19 pictures were scored fairly below the 25th percentile.

Spearman’s rho correlation revealed the degree of relationship among the four measures (Table 3). A significant negative correlation (*r*_*s*_ = – 0.99) between percentage and *H* statistic scores indicated that, overall, there was a good name agreement for most of the pictures in this set. A strong positive correlation (*r*_*s*_ = 0.83) was obtained between the scores of visual complexity and image agreement suggesting that sufficient details were incorporated to depict the verb pictures, which in turn, fostered their matching with the corresponding mental images created by the participants. The concept familiarity scores showed a moderate positive correlation with visual complexity (*r*_*s*_ = 0.56) and image agreement scores (*r*_*s*_ = 0.56). These suggested that familiar concepts were better accepted when depicted appropriately and vice versa. The visual complexity scores had a modest positive correlation (*r*_*s*_ = 0.30) with the percentage and a modest negative correlation (*r*_*s*_ = – 0.32) with the name agreement scores. These indicated that verb pictures are better identified when the right amount of detail is used to depict the target verbs and their corresponding argument structure(s). Overall, the one-argument verbs in this corpus (*n* = 85) are well developed, and are suitable for experiments, assessment and therapeutic protocols that need verbs as stimuli.

**Table 3:** Spearman’s rho correlation: one-argument verbs

	Name agreement		Word frequency	Concept familiarity	Visual complexity	Image agreement
	Percentage	*H* Statistic				
**Percentage**	1.00	– .991**	.008	.233*	.305**	.225*
***H***.**Statistic**		1.00	– .008	– .233*	– .320**	-.245*
**Word Frequency**			1.00	– .091	– .221*	-.122
**Concept Familiarity**				1.00	.562**	.566**
**Visual Complexity**					1.00	.832**
**Image Agreement**						1.00

** Correlation is significant at the 0.01 level (two-tailed).

* Correlation is significant at the 0.05 level (two-tailed).

## Two-argument verbs

This set contains the largest number of verbs (*n* = 132). Among these verbs (see Table 4 for the descriptive statistics), 93 received a perfect (Pct = 100%, *H* = 0) and 13 acquired a good name agreement score (Pct ≥ 85%, *H* ≤ 0.56). In the remaining verbs, the name agreement score of 24 ranged between 50% and 60% (Pct ≥ 60%, *H* ≤ 1.33), and that of the two was less than 50% score with an *H* statistic close to 1. The overall *H* statistic was positively skewed (1.42) with a low mean (M = 0.24; SD = 0.41) while the percentage scores were negatively skewed (– 1.88) with a higher mean (M = 92.92; SD = 12.91), indicating that a majority of the pictures within this set acquired a considerable name agreement. Ninety-eight pictures obtained a value lesser than the 75th percentile supporting this observation.

**Table 4:** Descriptive statistics: two-argument verbs

		Name Agreement		Word frequency	Concept familiarity	Visual complexity	Image agreement
		Percentage	*H* Statistic				
**Mean**		92.92	0.24	3.51	2.93	2.87	2.83
**Median**		100.00	0	2.00	2.93	2.93	2.93
**Standard Deviation**		12.91	0.41	4.86	0.07	0.16	0.19
**Minimum**		40.00	0	1.00	2.53	2.20	2.06
**Maximum**		100.00	1.36	34.00	3.00	3.06	3.00
**Range**		60.00	1.36	33.00	0.46	0.86	0.93
**Skewness**		– 1.88	1.42	3.52	– 1.84	– 1.43	-1.4
**Kurtosis**		3.02	0.62	15.19	4.776	1.90	2.25
**Percentile**	**25**	86.66	0	1.00	2.93	2.80	2.73
	**75**	100.00	0.56	3.00	3.00	3.00	3.00
**Interquartile Range**		13.33	0.56	2.00	0.06	0.20	0.26

Most of the concepts depicted in the two-argument set appeared familiar to the participants as the data was leptokurtic (4.77), and negatively skewed (– 1.84) with a high mean score (M = 2.93; SD = 0.07). However, 29 pictures scored marginally lower than the 25th percentile. The pictures seemed to be simple and filled with adequate details to most participants, as the data from visual complexity rating showed a higher mean (M = 2.87; SD = 0.16) with a platykurtic (2.80), and negatively skewed (– 1.43) pattern. Nonetheless, 27 pictures received a score lesser than the 25th percentile.

The participants seemed to agree that most of the pictures in this set were well developed with appropriate intricacies and mostly matched with the mental images of the target verbs. The image agreement data was platykurtic (2.25), and negatively skewed (– 1.40) along with a higher mean (M = 2.83; SD = 0.19). Predominantly, the pictures received ratings within the interquartile range (median = 2.93; IQR = 2.73–3.00). Yet, 27 pictures were scored fairly below the 25th percentile. There were no naming failures reported in this set of pictures.

Table 5 summarizes the association among the psychometric measures of the two-argument verb set. A strong negative correlation (*r*_*s*_ = – 0.99) between the percentage and *H* statistic indicated a good name agreement for most pictures within this set. A good positive correlation (*r*_*s*_ = 0.79) between visual complexity and image agreement ratings suggested that most verbs were well portrayed with the right amount of intricacies. They matched well with the participants’ mental image of the target verbs. Higher mean (M = 2.93; SD = 0.07) as well as a moderate correlation of concept familiarity with name agreement (*r*_*s*_ = 0.41; *r*_*s*_ = – 0.41), visual complexity (*r*_*s*_ = 0.48), and image agreement (*r*_*s*_ = 0.47) signified that this set contains homogenous and commonly used verbs that are well portrayed with adequate intricacies. A moderate correlation between image agreement and name agreement scores (IA and *Hr*_*s*_ = – 0.44; IA and Pct *r*_*s*_ = 0.44) reinforces this observation and indicates that well-depicted images have better imageability, and are effortlessly identified.

**Table 5:** Spearman’s rho correlation: two-argument verbs

	Name agreement		Word frequency	Concept familiarity	Visual complexity	Image agreement
	Percentage	*H* Statistic				
**Percentage**	1.00	– .997**	– .171	.418**	.357**	.445**
***H***.**Statistic**		1.00	.175*	– .410**	– .360**	-.448**
**Word Frequency**			1.00	.011	-.018	-.102
**Concept Familiarity**				1.00	.485**	.478**
**Visual Complexity**					1.00	.790**
**Image Agreement**						1.00

** Correlation is significant at the 0.01 level (two-tailed).

* Correlation is significant at the 0.05 level (two-tailed).

## Three-argument verbs

Like the previous sets (see Table 6 for the descriptive statistics), among the verbs in three-argument group (*n* = 52), 33 acquired a perfect name agreement score (Pct = 100%, *H* = 0). Five verbs received a good name agreement (Pct ≥ 85%, *H* ≤ 0.56), 12 yielded moderate name agreement (Pct ≥ 60%, *H* ≤ 1.24), and the remaining two obtained a fair score (Pct ≤ 53.33%, H ≥ 0.99). Primarily, the *H* statistic was positively skewed (1.31) with a low mean (M = 0.31; SD = 0.47), whereas the percentage scores were negatively skewed (– 1.91) with a higher mean (M = 90.32; SD = 15.90), signifying a considerable name agreement for most pictures within this set. Furthermore, 38 pictures attained an *H* statistic lesser than the 75th percentile supporting this observation.

**Table 6:** Descriptive statistics: three-argument verbs

		Name agreement		Word frequency	Concept familiarity	Visual complexity	Image agreement
		Percentage	*H* Statistic				
**Mean**		90.32	0.31	3.88	2.92	2.85	2.77
**Median**		100.00	0.00	2.00	2.93	2.93	2.86
**Standard Deviation**		15.90	0.47	4.43	0.07	0.17	0.22
**Minimum**		26.66	0.00	1.00	2.73	2.33	2.13
**Maximum**		100.00	1.88	21.00	3.00	3.00	3.00
**Range**		73.33	1.88	20.00	0.26	0.66	0.86
**Skewness**		– 1.91	1.31	2.20	– 1.08	– 1.3	-1.12
**Kurtosis**		4.11	1.04	5.27	0.87	1.2	0.55
**Percentile**	**25**	80.00	0.00	1.00	2.86	2.80	2.60
	**75**	100.00	0.72	6.00	3.00	3.00	2.93
**Interquartile range**		20.00	0.72	5.00	0.13	0.20	0.33

A negatively skewed (– 1.84) data with a high mean score (M = 2.92; SD = 0.07) suggests that the participants were mostly familiar with the verbs in this set, and only six pictures scored marginally lower than the 25th percentile. The verb pictures in this set are also rated to have been portrayed with adequate details as the data from visual complexity rating showed a higher mean (M = 2.85; SD = 0.17) and a negatively skewed (– 1.12) pattern. However, ten verbs received a marginally lesser score than the 25th percentile.

The image agreement data was negatively skewed (– 1.40) with a higher mean (M = 2.83; SD = 0.19), suggesting that most pictures represented the verbs and their corresponding arguments adequately in the target sentences. Further, in the set, more than half of the pictures (i.e., 29/52) seemed to match well with the participants’ mental image, as their image agreement scores fell within the interquartile range (median = 2.86; IQR = 2.60-2.93). However, ten pictures were scored fairly below the 25th percentile. There were no naming failures reported in this set of pictures.

Table 7 summarizes the correlation between the psychometric measures of the three-argument verbs set. A strong negative correlation (*r*_*s*_ = – 0.99) between the percentage and *H* statistic indicated a good name agreement for the majority of the pictures within this set. A good positive correlation (*r*_*s*_ = 0.88) between visual complexity and image agreement suggested that most verbs were well portrayed with the right amount of intricacies. They matched well with the participants’ mental image of the target verbs.

**Table 7:** Spearman’s rho correlation: three-argument verbs

	Name agreement		Word frequency	Concept familiarity	Visual complexity	Image agreement
	Percentage	*H* Statistic				
**Percentage**	1.00	– .995**	.078	.243	.362**	.241
***H***.**Statistic**		1.00	– .089	– .275	– .380**	-.261
**Word frequency**			1.00	– .052	– .093	-.031
**Concept familiarity**				1.00	.663**	.562**
**Visual complexity**					1.00	.885**
**Image agreement**						1.00

** Correlation is significant at the 0.01 level (two-tailed).

* Correlation is significant at the 0.05 level (two-tailed).

In this study, a linguistically and culturally apposite set of 269 verb picture corpus was developed. Normative data was established in Kannada based on four psychometric parameters: verb name and argument agreement, image agreement, concept familiarity, and visual complexity. This dataset is a first of its kind in an Indian language as the verb pictures are developed with their argument structure in the sentence contexts. Norms collected from 120 adult native Kannada-speakers (*Kannadigas*) belonging to four major dialects are described. The database also includes corresponding orthographic word frequency scores extracted from a published repository (Ranganatha, 1982).

Initially, 293 verb pictures were utilized to collect the normative for verb name and argument agreement tasks. Pointers were incorporated in a few pictures to prime the agent to the participants. This was done to prevent ambiguity and role reversals especially for those pictures that involved the identification of an action amidst two animate objects. For example, the picture number 102 was designed to portray a man buying vegetables from a woman. Without a pointer on the man, this picture could unintentionally be interpreted as a lady selling vegetables to the man.

Twenty-four images were excluded from further ratings after obtaining dominant responses that were completely different from the intended target verb. Overall, the scores were high with negligible naming failures. Yet, in this dataset, a higher variability was observed in the participants’ responses in comparison to the noun database of Snodgrass and Vanderwart (1980). As discussed previously, this variability can be attributed to the conceptual organization of verbs, especially when a wide variety of actions and events are depicted within a corpus (Fiez & Tranel, 1997). Verbs tend to have a larger variance in name agreement scores as they rarely exist independently, unlike nouns (Fiez & Tranel, 1997; Khwaileh et al., 2018). Verbs are more complexly represented with lower semantic richness in comparison to nouns, which can influence naming accuracy and latency (Mätzig et al., 2009). We classified the responses as synonymous if the verbs could be interchangeably used to describe an action or event (Fiez & Tranel, 1997).

For example, colloquially, the verbs  (To kill someone/something) can be interchangeably used within the sentence

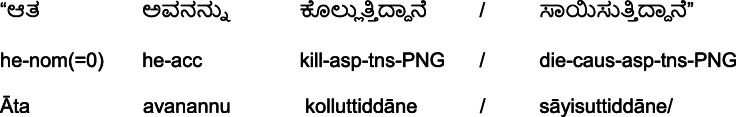



Similarly, the verbs  (To attract someone/something) can be used interchangeably in the sentence

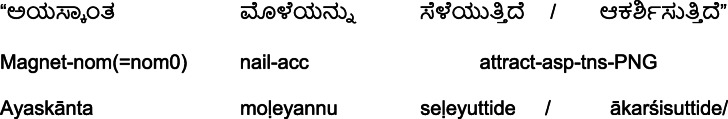



Furthermore, verbs that represented different exemplars of the same category (coordinate verbs) were portrayed as separate images altogether (Fiez & Tranel, 1997).

For example, consider the following two sentences:

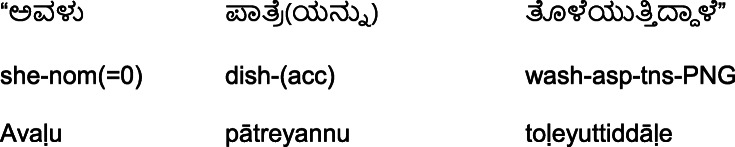



meaning “A lady is washing the dishes” and

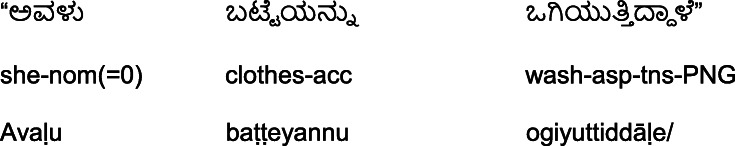



meaning “She is washing the clothes,”

here the verbs  /ogi/ both represent the act of washing or cleaning something. Yet, they can be considered as separate verbs as they colloquially describe two separate actions, and using them interchangeably will render the sentences syntactically inappropriate. Thus, we decided not to treat such synonymous and coordinate verbs as naming failures or disagreement, unlike Fiez and Tranel (1997).

The final set of 269 verb pictures (one-argument verbs = 85; two-argument verbs = 132; three-argument verbs = 52) were presented to the participants for rating concept familiarity, visual complexity, and mage agreement. Overall, the results indicate that the verbs included in this corpus have a substantial name and argument agreement. The corpus includes predominantly familiar concepts that are mostly portrayed with sufficient visual details.

The findings from this study are in accordance with the results of previous similar studies that intended to develop standardized verb picture corpora. A higher verb name and argument agreement score ensures greater accuracy in naming the target verbs and corresponding arguments. Naming accuracy and latency seem to increase when a target concept has lesser competing lexical items.

The good correlation between verb name agreement and image agreement observed in this dataset implies that whenever the displayed target image matches with the participant’s mental image, the verb and their corresponding argument(s) are named with low variability (Akinina et al., 2015; Bonin et al., 2004; Shao et al., 2014). Participants tend to possess a typical mental image for each verb, and when the target verb picture matches with it, the probability of consistently naming the target is higher (Akinina et al., 2015). These typical mental images may promote easier imageability, and when a verb evokes a specific mental image, the chances of it being named in the same manner become higher (Akinina et al., 2015; Barry et al., 1997; Bonin et al., 2004; Khwaileh et al., 2018; Shao et al., 2014). The good correlation between the two measures also indicates that the verb pictures and their corresponding exemplar sentences in this dataset are suitable for use with participants across different dialects of Kannada.

The participants found most of the verbs in this data set familiar, and reported to have experienced them frequently in their daily life. They concurred that majority of the verbs were befittingly depicted using the right amount of details. A strong correlation between concept familiarity and visual complexity ratings supports this observation (Akinina et al., 2015; Bonin et al., 2004; Fiez & Tranel, 1997).

A positive correlation between concept familiarity and image agreement suggests that participants could effortlessly generate mental images for familiar verbs, which can be attributed to the availability of typical mental images (Akinina et al., 2015). Image agreement significantly correlated with visual complexity throughout this data set, indicating that most verbs have been portrayed with adequate details and matched well with the participants’ mental image with ease (Akinina et al., 2015; Shao et al., 2014).

### Dialectal and colloquial variances

Kannada has four major dialects and multiple sub-dialects(Steever, 2003, p. 129; Karnataka Government, 2020). The target sentences in this dataset can be represented variably in different dialects. For instance, consider the sentence “The boy is running”:

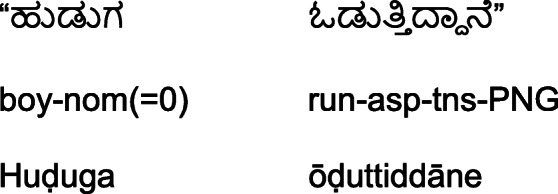



Here, this sentence is represented in a standard version of Kannada, which is commonly used in textbooks, administrative, and multimedia communications. In the colloquial form, the same sentence is commonly exemplified as:

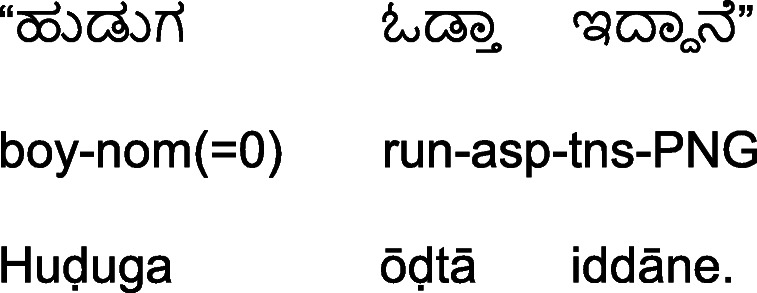



In the northern Karnataka dialect, this can be represented as:

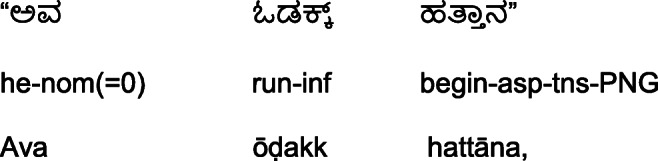



or represented as

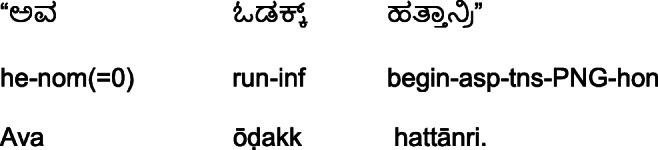



However, in the rural dialects spoken in and around Mysore district (the old capitol of Karnataka),

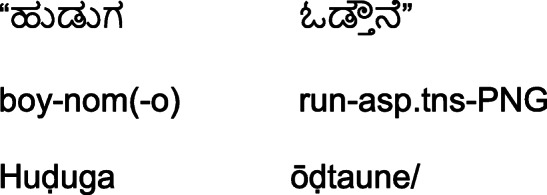



is a more common form.

It is, therefore, unrealistic to document all dialectal versions of the dominant (verb name and argument agreement) responses within the realm of this study. Thus, for the ease of understanding and cross referencing, we have presented only the standard version of the sentences in this database (see Appendix).

A majority of the sentences in this database begin with  /Avanu/ (He) and  /Avaḷu/ (She) rather than beginning with  /Huḍuga/ (The boy) or  /Huḍugi/ (The girl). This can be found in colloquial usage across dialects, especially while addressing an adult man or woman, because addressing someone as  manuṣya/ (That Man) or  (That lady) can be considered culturally offensive. All these responses were commonly provided by the participants hailing from different dialectal backgrounds.

Variances in suffixal usage can also be found in this dataset. In a given sentence, the suffixes /ḷe/ and /ne/ are used to refer to a female and male subject, respectively, in singular form, whereas the suffix /re/ is commonly used for plural form of the subject.

For example:

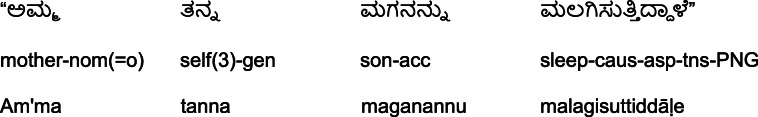



meaning “The mother is putting her son to sleep”.

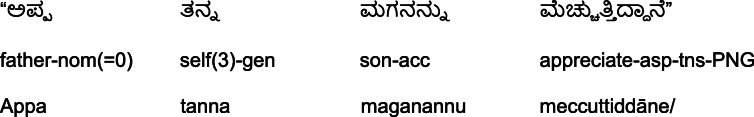



meaning “The father is admiring his son”

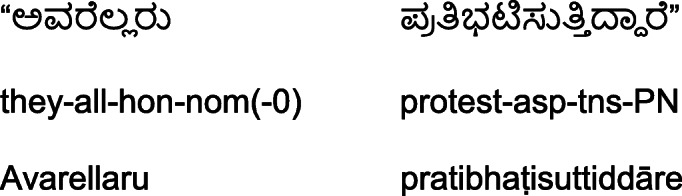



meaning “They all are protesting”. Yet, /re/ is also colloquially used as a mark of respect while addressing to an authoritative or elderly subject.

For instance:

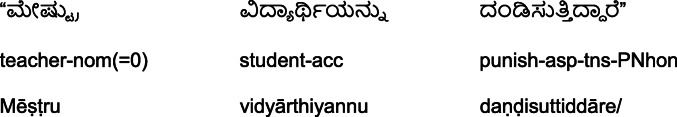



meaning “The teacher is punishing the student”

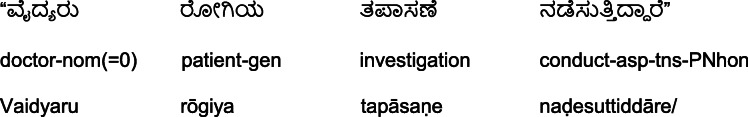



meaning “The doctor is examining the patient”

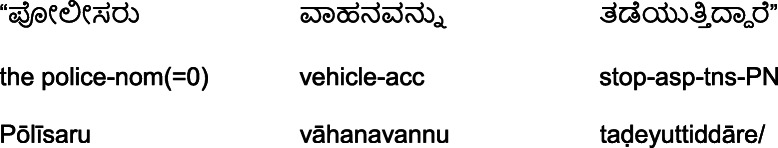



meaning “The policeman is stopping the vehicle”

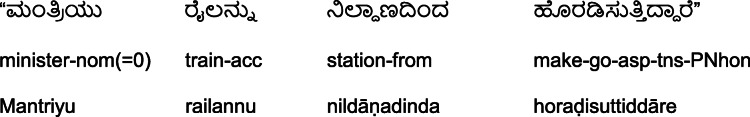



meaning “The minister is flagging off the train from the station.”

In colloquial usage, the English version of nouns and verbs can also be found (Ahmed et al., 2008). For example, in the following sentence:

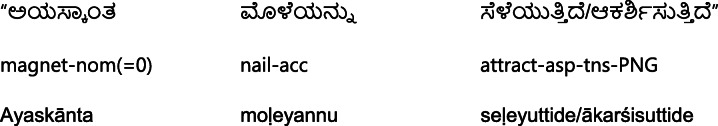



meaning “The magnet is attracting the nail,”

the English version "magnet" might be more colloquially used than the Kannada version /Ayaskānta/. Thus, users of this dataset are given the liberty to either adapt, accept, or reject the dialectal, and colloquial variations based on their experimental/practical requirements.

A few observations from this study are in contrast to Fiez and Tranel (1997), as every verb in this set was depicted as an independent single image, and a comparison between two images to infer an action was unnecessary. Predominantly, we intended to represent the verbs in present tense. However, five pictures received dominant response in the past tense (see picture nos. 37, 73, 81, 99 & 194: Appendix). We envisioned to incorporate various agent types (person, animal, or object) performing the actions, thus, representing a broad variety of stimuli with diverse thematic roles and argument structures (Fiez & Tranel, 1997).

Animate nouns form a dominant portion of this dataset, yet, inanimate nouns were represented as agents in some pictures (# 2, 19, 29, 47, 49, 73, 77, 78, & 81 for one-argument verbs, # 99, 140, & 153 for two-argument verbs, and # 244 for three-argument verbs, respectively). Furthermore, the 269 verbs in this dataset have been classified based on the obligatory and optional argument structure for the ease of the users to select the pictures. The details are provided as supplementary list, and can be accessed at: https://osf.io/uk2af/?view_only=ecffbd92f48546a484c869b3f0b8ec94 link.

The development and validation of verbs or action picture corpora is certainly a meticulous process. A mere translation of a database, especially in the case of verbs, developed in another language may not necessarily be appropriate. Yet, such studies are valuable when they are readily accessible to clinicians and researchers alike. In this study, a sincere attempt has been made to provide such a useful corpus for the adult *Kannadiga* population, a first of its kind among the Indian languages. Furthermore, this dataset is unique as it gives norms for verb pictures that have been depicted based on exemplar sentences. The verbs are depicted contextually in order to portray the target actions and events with a diversity of thematic roles (Fiez & Tranel, 1997).

Predominantly, the existing verb corpora offer normative data for action naming alone, and the associated argument structure details need to be accessed from other repositories, or the users have to classify the verbs on their own. The unavailability of such a repository in Kannada restricts the investigators from designing quality experiments that need verbs as stimuli. Further, the corpus is expected to contribute to the assessment and management of grammatical impairments in Kannada-speaking persons with aphasia as well as in children with language (grammatical) impairments. The outcome from this study is expected to fill this gap, and pave the way for more such development and validation studies in other Indian languages as well as many less explored languages across the world.

## Conclusions

This study discusses the details of the development and validation of a large set of (269) verbs with their argument structures, a type of verb corpus seldom found in the literature, akin to the landmark study by Snodgrass and Vanderwart (1980). This effort is expected to stimulate similar such studies in other languages, thus furthering our understanding of verb argument processing in various populations of interest, including persons with aphasia and other cognitive-linguistic deficits such as dementia, children with language impairments, aging adults, to name a few.

## Data Availability

All data generated or analyzed during this study are included in this published article (and its supplementary information files). The standardized pictures are available on the link -https://osf.io/uk2af/?view_only=ecffbd92f48546a484c869b3f0b8ec94
